# Transcriptome Profiling of the Hippocampal Seizure Network Implicates a Role for Wnt Signaling during Epileptogenesis in a Mouse Model of Temporal Lobe Epilepsy

**DOI:** 10.3390/ijms231912030

**Published:** 2022-10-10

**Authors:** Muriel D. Mardones, Kunal Gupta

**Affiliations:** 1 Stark Neurosciences Research Institute, Indiana University School of Medicine, Indianapolis, IN 46202, USA; 2 Department of Neurological Surgery, Indiana University School of Medicine, Indianapolis, IN 46202, USA

**Keywords:** hippocampus, mechanisms, adult hippocampal neurogenesis, translational research, epilepsy, Wnt signaling, neuroinflammation

## Abstract

Mesial temporal lobe epilepsy (mTLE) is a life-threatening condition characterized by recurrent hippocampal seizures. mTLE can develop after exposure to risk factors such as febrile seizure, trauma, and infection. Within the latent period between exposure and onset of epilepsy, pathological remodeling events occur that contribute to epileptogenesis. The molecular mechanisms responsible are currently unclear. We used the mouse intrahippocampal kainite model of mTLE to investigate transcriptional dysregulation in the ipsilateral and contralateral dentate gyrus (DG), representing the epileptogenic zone (EZ) and peri-ictal zone (PIZ). DG were analyzed after 3, 7, and 14 days by RNA sequencing. In both the EZ and PIZ, transcriptional dysregulation was dynamic over the epileptogenic period with early expression of genes representing cell signaling, migration, and proliferation. Canonical Wnt signaling was upregulated in the EZ and PIZ at 3 days. Expression of inflammatory genes differed between the EZ and PIZ, with early expression after 3 days in the PIZ and delayed expression after 7–14 days in the EZ. This suggests that critical gene changes occur early in the hippocampal seizure network and that Wnt signaling may play a role within the latent epileptogenic period. These findings may help to identify novel therapeutic targets that could prevent epileptogenesis.

## 1. Introduction

Epilepsy is a common neurological disorder that afflicts 1–2% of the U.S. population; the most common form of adult focal epilepsy is mesial temporal lobe epilepsy (mTLE), which is medically refractory in 40% of patients [1,2,3,4]. In many cases, an inciting event or risk factor (e.g., febrile seizure, trauma, infection) can be identified prior to the onset of clinical epilepsy [5,6,7,8,9]. The process of epileptogenesis, which occurs in this latent period, is characterized by pathological remodeling of the hippocampus, specifically the dentate gyrus. Critically, rodent models of epilepsy recapitulate important aspects of human mTLE, such as mesial temporal sclerosis, hippocampal dentate granule cell dispersion, dentate granule cell mossy fiber sprouting, and the loss of hippocampal hilar and pyramidal neurons [10,11,12,13]. Furthermore, induction of a single episode of status epilepticus (SE) by a variety of established methods in these rodent models typically results in acquisition of epilepsy within two weeks [14,15,16], affording a unique opportunity to investigate the earliest stages of epileptogenesis in vivo.

It is increasingly well recognized that the epileptic brain develops established seizure propagation pathways that predispose one to recurrent seizures [17,18,19], and persistence of these epileptic circuits after surgery may be responsible for delayed surgical failures [20,21]. The molecular mechanisms responsible for epileptogenesis and seizure network formation represent unique therapeutic targets that might prevent epileptogenesis after a neurological insult and may even improve the efficacy of clinical interventions. As a result, prior studies have sought to investigate transcriptomic and proteomic changes in human and animal models of epilepsy [22,23,24,25,26,27]; several studies have indeed focused on the dentate gyrus as one of the key sites in mTLE epileptogenesis [22,23,26]. These studies and others have contributed significantly to our understanding of epilepsy-specific gene expression changes, implicating specific molecular pathways including MAPK, NMDA-receptor-mediated excitotoxicity, CaMK, mTor, and Wnt [22,25,27,28]. These previous studies, however, have not examined differences between the ictal/epileptogenic zone and peri-ictal regions of the seizure network at serial time-points during epileptogenesis.

There is growing evidence that the Wnt pathway may provide some of the key early signaling events during epileptogenesis [29,30,31,32]. The Wnt pathway encompasses a family of 19 secreted Wnt protein ligands, which bind a family of 10 membrane frizzled receptors and co-receptors; these signal to downstream canonical beta-catenin-dependent and non-canonical planar cell polarity and calcium pathways. The canonical Wnt pathway is activated by the binding of Wnt ligands to a frizzled receptor and the LRP5/6 co-receptor; this results in recruitment of the beta-catenin destruction complex to the plasma membrane, allowing nuclear translocation of beta-catenin and activation of transcription factors TCF/LEF1. The planar cell polarity (PCP) non-canonical Wnt pathway signals through c-Jun N-terminal kinase (JNK) via Rho and Rac GTPases, and the calcium non-canonical Wnt pathway results in the activation of calmodulin-dependent protein kinase II (CaMKII), calcineurin, and protein kinase C (PKC), via phospholipase C signaling [33]. Under physiological conditions, Wnt signaling has been implicated in neurogenesis and dendrite formation in the adult rodent hippocampus [34,35,36,37,38]. More recently, the canonical Wnt pathway has also been implicated under pathophysiological conditions during epileptogenesis, affecting pro-epileptogenic neuronal remodeling and neurogenesis in the hippocampal dentate gyrus [30,39,40]. As yet, the specific role of Wnt signaling in epileptogenesis remains to be determined.

In this study, we used the adult mouse intrahippocampal kainate model of focal mesial temporal lobe epilepsy and performed transcriptome profiling of the hippocampal dentate gyrus. Our use of this model provides a unique opportunity to examine transcriptional dysregulation in varying regions of the seizure network. It has been demonstrated that the contralateral dentate gyrus also undergoes pathological remodeling in this model, and it is well recognized that the contralateral hippocampus is an independent source of epileptic seizures [30,41]. We therefore investigated both the ipsilateral kainate-injected dentate gyrus, representing the epileptogenic zone, and the contralateral dentate gyrus, representing the peri-ictal zone, recognizing the role of both parts of the seizure network in maintaining epilepsy [17,18,19,30]. We selected time-points during epileptogenesis, 3 days, 7 days, and 14 days after SE, which fall within the latent period between SE and the onset of spontaneous recurrent seizures [15,22,23]. The data presented herein identify distinct patterns of differential gene expression within the seizure network, which are dynamic over the early epileptogenic period: In the epileptogenic zone, we identified early enrichment of cell signaling and delayed inflammatory processes, and in the peri-ictal zone we identified a brief early enrichment of cell signaling and inflammatory processes. We further observed that Wnt signaling was dysregulated during early epileptogenesis in both the epileptogenic zone and the peri-ictal zone. These findings provide clues as to the key dysregulatory events that may contribute to epileptogenesis, which may potentially help identify novel therapeutic targets that might be modulated to prevent the development of epilepsy.

## 2. Results

### 2.1. Mesial Temporal Lobe Epilepsy Model and Experimental Design

**Figure 1:**
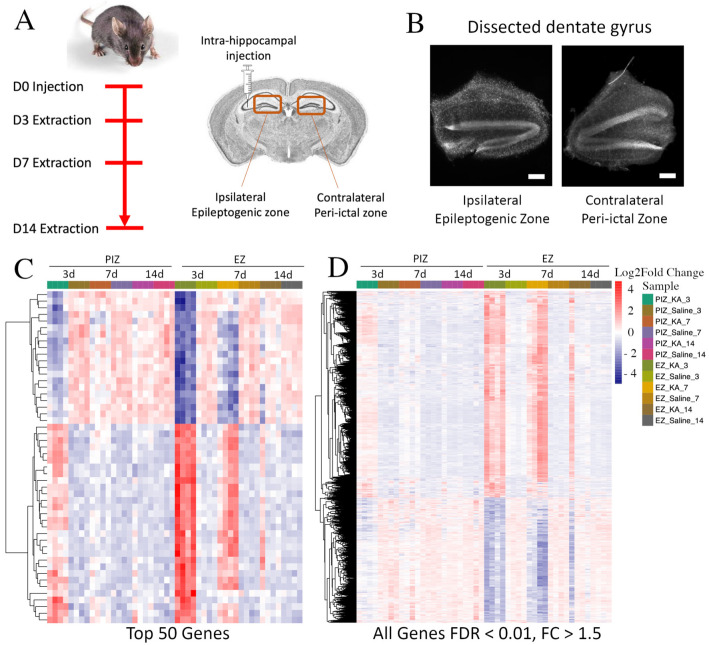
RNA sequencing of the ipsilateral and contralateral hippocampal dentate gyri during epileptogenesis. (**A**) Timeline and schematic of the experimental design. Mice were injected unilaterally in the hippocampus with kainate to induce SE or with saline control and sacrificed 3 days, 7 days, and 14 days after injection. The bilateral dentate gyri were harvested, and the dorsal portions underwent RNA sequencing. (**B**) Representative DAPI-stained brightfield coronal sections of anatomically dissected ipsilateral and contralateral dentate gyrus; scale bar 100 µm. Hierarchical cluster analysis demonstrating (**C**) the top 50 genes and (**D**) genes across the entire transcriptome with FDR < 0.01 and FC > ±1.5 dysregulated in the EZ and PIZ 3 days after SE induction across all samples. *n* = 4 mice per experimental group (2 males and 2 females).

In the intra-hippocampal kainate model of epileptogenesis, spontaneous recurrent seizures occur 10–14 days after SE-induction [15,16]. We therefore hypothesized that critical gene expression changes that may be responsible for the development of epilepsy may occur within this latent period. To identify differentially expressed genes (DEG) at the earliest stages of epileptogenesis, we focused our investigation on the first 2 weeks after SE induction (Figure 1A). Since the dentate gyrus has been specifically implicated in temporal lobe epilepsy [10,11,12,13,42], we performed anatomical microdissection and extracted the dentate gyrus from kainate and saline-vehicle-control-injected animals for analysis as previously described [30] (Figure 1B). Time-points sampled were 3 days, 7 days, and 14 days, representing the end of SE, the period between SE and the onset of recurrent seizures, and the period near the onset of recurrent seizures, respectively [16]. It has been shown previously that immature dentate granule cells in the contralateral dentate gyrus also undergo remodeling 2 weeks after SE and, as a result, may contribute to the maintenance of the seizure network [17,18,19,30]. Therefore, transcriptional dysregulation in both the ipsilateral dentate gyrus epileptogenic zone (EZ) and the contralateral dentate gyrus peri-ictal zone (PIZ) were assessed (Figure 1A), encompassing different regions of the seizure network.

### 2.2. Transcriptomic Dysregulation across the Seizure Network in Early Epileptogenesis

We initially examined the relative expression of differentially expressed genes between kainate-injected epilepsy animals and saline-injected control animals between 3 to 14 days after induction of status epilepticus. In doing so, we were able to broadly assess the overall patterns of gene dysregulation over the first 2 weeks of epileptogenesis associated with both the epileptogenic zone and the peri-ictal zone. The gene expression heatmap demonstrates that in the epileptogenic zone, within the top 50 DEG, 60% were upregulated (30/50) and 40% were downregulated (20/50), 3 days after SE induction (Figure 1C). This pattern was maintained, although with an overall reduction in fold-change magnitude, in the epileptogenic zone 7 days after SE induction and began to normalize 14 days after SE induction. When we assessed the peri-ictal zone, we observed a similar pattern of differential expression in the same subset of genes that were dysregulated in the epileptogenic zone 3 days after SE induction, although at an overall reduced fold-change magnitude. The magnitude of dysregulation of these top 50 genes in the peri-ictal zone 3 days after SE-induction was comparable to the levels observed 7 days after seizure induction in the epileptogenic zone. In the peri-ictal zone, gene expression changes began to subside at 7 days and were normalized by 14 days after SE induction when compared to saline controls. These patterns of gene expression were also observed across the entire transcriptome (Figure 1D), and when assessed at the other sampled time points (Figure S1).

These data suggest that unique transcriptional dysregulation events occur within the first 2 weeks after SE induction; these may be important in the development of spontaneous recurrent seizures, which occur toward the end of this latent period. These data also suggest that some transcriptional changes are shared between the epileptogenic zone and seizure network, and that transcriptional dysregulation is of greater magnitude and prolonged in the epileptogenic zone.

### 2.3. Shared Patterns of Gene Dysregulation between the Epileptogenic Zone and Peri-Ictal Zone across Early Epileptogenesis

**Figure 2:**
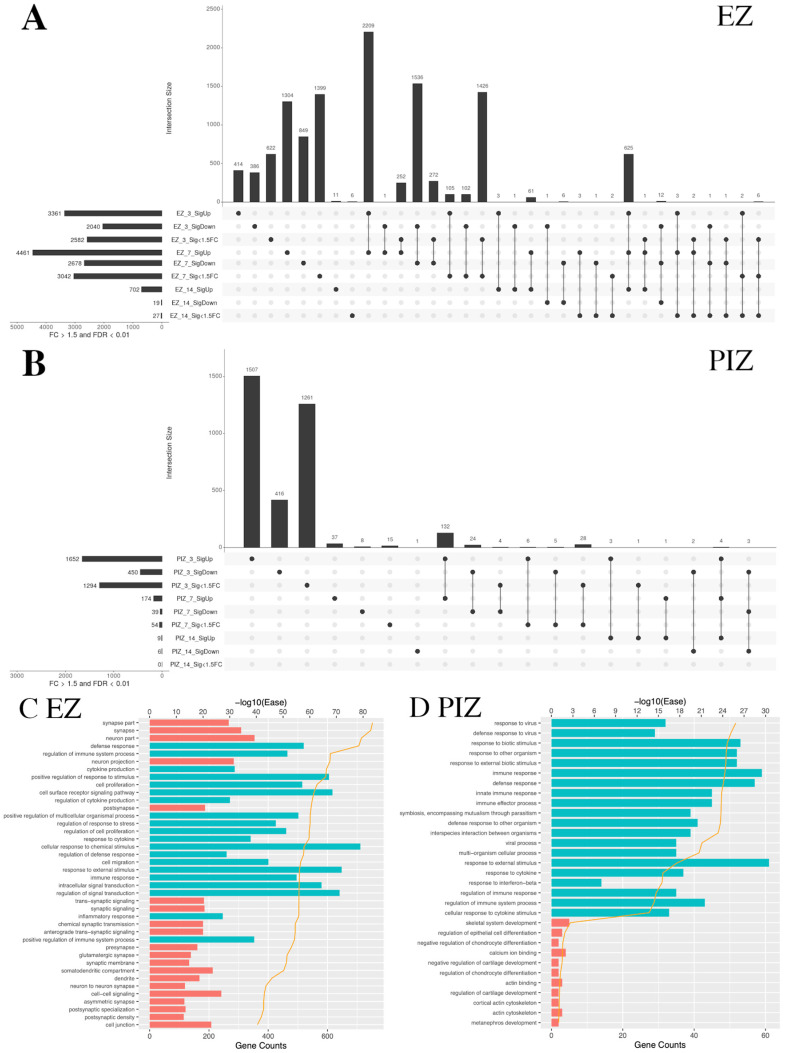
UpSet plots depicting the extent of shared transcriptional dysregulation in the EZ and PIZ across the first 2 weeks of epileptogenesis. In each UpSet plot, the vertical bars represent gene number for the corresponding group/overlapping groups. Below the histogram, the circle-line chart shows the group depicted for each bar; individual dots denote genes specific to an individual group, and linked dots represent overlapping gene groups. The horizonal bars denote total gene number for each region/time-point/differential expression group. Significantly upregulated genes are labeled as SigUp, significantly downregulated genes are labeled as SigDown, and genes with FDR < 0.01 but with less than 50% fold change are labeled as “Sig < 1.5 FC”. (**A**) Overlapping gene groups in the epileptogenic zone 3, 7, and 14 days after seizure induction. (**B**) Overlapping gene groups in the peri-ictal zone 3, 7, and 14 days after seizure induction. (**C**) Top 20 upregulated and dysregulated DAVID gene ontology terms in the EZ shared between the 3-day and 7-day time-points. (**D**) Top 20 upregulated and dysregulated DAVID gene ontology terms in the PIZ shared between the 3-day and 7-day time-points. Tables listing names for the genes represented in each UpSet plot can be found in Tables S1 and S2.

We next examined overlapping gene groups across the first 2 weeks of epileptogenesis in the EZ and PIZ. Data are represented as UpSet plots; total gene number in each individual and overlapping group is represented by the histogram, and the groups included are denoted by the circle-line plot beneath each bar of the histogram. Over early epileptogenesis in the EZ, the largest overlapping groups of DEG were observed between 3 days and 7 days after SE induction. This indicates a large degree of overlap in underlying gene dysregulation, and therefore shared molecular processes, during the first week after SE in the epileptogenic zone. The number of DEG that remain either upregulated or downregulated 14 days after SE induction fell by 95%, suggesting that the critical period after SE may be captured within the first week (Figure 2A, Table S1). By comparison, in the peri-ictal zone, the majority of genes were uniquely dysregulated 3 days after SE induction, with few dysregulated genes observed at subsequent time-points; we observed less overlap between the 3-day and 7-day time-points, with overlap near-negated at the 14-day time-point (Figure 2B, Table S2). These data suggest that the principal gene dysregulation period in the peri-ictal zone may occur within 3 days of SE induction. 

We therefore examined the top 20 upregulated and downregulated DAVID (Database for Annotation, Visualization and Integrated Discovery) pathway gene ontology (GO) categories shared across the first 7 days after SE induction in the EZ and PIZ. In the EZ, we observed upregulation of GO terms associated with cell signaling and remodeling, including “cell proliferation”, “cell surface receptor signaling pathway”, “cell migration”, and “signal transduction”, as well as inflammatory terms such as “cytokine production” and “defense response” (Figure 2C). These suggest a prolonged period of cell-signaling events for 7 days after SE induction that may encompass epileptogenic molecular processes. As expected, due to the extensive neuronal loss that occurs in the epileptogenic zone, neuronally associated GO terms such as “synapse” and “dendrite” were among the top 20 downregulated GO terms. In the PIZ, the top 20 upregulated GO terms shared across the first week of epileptogenesis were associated predominantly with inflammation (Figure 2D).

When we compared across regions at each time-point, the epileptogenic zone and peri-ictal zone demonstrated different patterns of gene dysregulation. Three days after SE induction, there were 1389 overlapping genes between significantly upregulated and downregulated genes in the epileptogenic zone and peri-ictal zone (Figure S2A, Table S3). This suggests that some gene dysregulation events and their corresponding pathways are shared between these two regions of the seizure network 3 days after SE induction. However, the number of overlapping genes was reduced at 7 days (Figure S2B, Table S4) and at 14 days (Figure S2C, Table S5) after SE induction, suggesting that gene dysregulatory events diverge beyond 3 days after SE. Fourteen days after SE, 702 genes were upregulated in the epileptogenic zone, 699 uniquely, and only 3 were shared with the peri-ictal zone (Figure S2C); however, 691 genes overlapped with earlier time-points in the epileptogenic zone 3 days and 7 days after SE (Figure 2A). These data indicate that transcriptional dysregulation in the peri-ictal zone declines 14 days after SE induction, and the epileptogenic zone retains persistent gene dysregulation, as might be expected from the extent of injury and remodeling observed in the epileptogenic zone. Gene lists for each overlapping group are listed in Tables S1–S5.

### 2.4. Enrichment of Functional DAVID Categories in the Epileptogenic Zone and Seizure Network across Early Epileptogenesis

**Figure 3:**
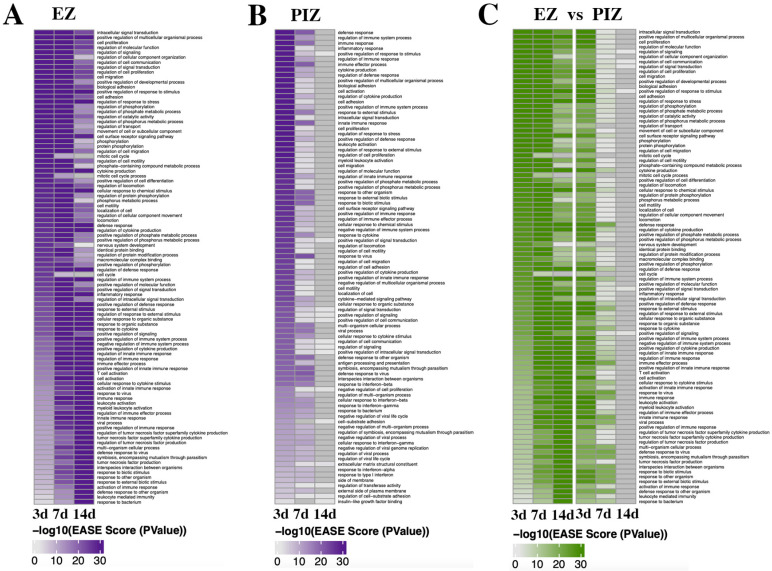
Gene Ontology term enrichment in the EZ and PIZ over the first 2 weeks of epileptogenesis. Significant DAVID pathway gene ontology terms are expressed over the first 2 weeks of epileptogenesis as heatmaps across the EZ and PIZ. (**A**) Enrichment of GO terms in the EZ, 3 days, 7 days, and 14 days after SE induction. (**B**) Enrichment of GO terms in the PIZ, 3 days, 7 days, and 14 days after SE induction. (**C**) Depiction of GO term enrichment in the PIZ of the most highly enriched GO terms in the EZ, demonstrating how GO terms enriched in the EZ are represented in the PIZ across the first 2 weeks of epileptogenesis.

In order to investigate the functional effect of transcriptional dysregulation in epileptogenesis across the two regions, we examined the enrichment of gene ontology (GO) terms within DAVID pathways. We specifically identified the top 50 most significantly enriched GO categories within each of the two regions at each time point (Figure 3A,B). We also compared the pattern of expression in the peri-ictal zone with the top-most highly enriched categories in the epileptogenic zone (Figure 3C). We observed that in the epileptogenic zone, different overall patterns of GO terms were shared between the 3- and 7-day time-points, compared to between the 7- and 14-day time-points, with a number of GO terms unique to the 14-day time-point. In the epileptogenic zone, GO terms enriched at 3 days and 7 days after SE were associated with cell signaling (e.g., intracellular signal transduction, regulation of signaling, regulation of cell communication, regulation of signal transduction, positive regulation of molecular function, positive regulation of signal transduction, regulation of intracellular signal transduction), cell migration and motility (e.g., cell migration, cell adhesion, movement of cell or subcellular component, regulation of cell motility, regulation of locomotion, locomotion, cell motility, localization of cell), and cell survival/proliferation (e.g., cell proliferation, neurogenesis, cell death, apoptotic process, programmed cell death, cell development) (Figure 3A). These GO terms encompass pathological cellular processes observed in the dentate gyrus after SE [43] and suggest that the molecular mechanisms responsible for these pro-epileptogenic processes may be represented in this dataset and may occur early after SE. GO terms enriched at the 7-day and 14-day time-points, as well as more prominently in the 14 day time-point, are related to inflammatory processes (e.g., immune response, response to cytokine, inflammatory response, regulation of TNF superfamily cytokine production, regulation of adaptive immune response and more). These inflammatory GO terms are less represented at the 3-day time-point, and if present, overlap with the 7-day and 14-day time-points. This suggests that pro-inflammatory processes occur in a delayed and prolonged manner from 7 days after SE in the epileptogenic zone.

Within the peri-ictal zone, the majority of the most highly enriched GO terms were represented uniquely at 3 days or 7 days after SE induction with few overlapping groups; none were represented at 14 days (Figure 3B). These data further support the return of transcriptional dysregulation to baseline 14 days after SE induction. The GO terms enriched 3 days after SE induction were, similarly to the 3-day/7-day time-point in the epileptogenic zone, associated with cell signaling (e.g., intracellular signal transduction, positive regulation of signal transduction, cell surface receptor signaling pathway, regulation of signal transduction, regulation of cell communication, positive regulation of cell signaling), cell migration and motility (e.g., cell adhesion, cell migration, regulation of cell motility, regulation of locomotion, positive regulation of cell adhesion), and cell survival/proliferation (e.g., cell proliferation, regulation of cell proliferation, positive regulation of developmental process), although with less representation of cell-death-related GO terms, as might be expected due to the lower cell death observed in comparison the ipsilateral epileptogenic zone [30]. There is, however, earlier representation of inflammation-related GO terms (e.g., regulation of immune system process, immune response, cytokine production, leukocyte activation, regulation of inflammatory response) 3 days after SE induction, which also comprise the predominantly represented terms 7 days after SE in the peri-ictal zone (e.g., response to cytokine, cellular response to interferon gamma, antigen processing and presentation of peptide antigen). These data suggest that similar alterations in molecular signaling may also drive epileptogenic remodeling in the peri-ictal zone seizure network.

When we examined how the most highly enriched GO terms in the epileptogenic zone were expressed in the PIZ across the 3-, 7-, and 14-day time-points (Figure 3C), we observed that there were similar functional consequences between the ipsilateral and contralateral regions 3 days after SE induction. We observed enrichment of GO terms associated with cell signaling (e.g., intracellular signaling induction, regulation of signaling, regulation of cell communication, regulation of molecular function, regulation of signal transduction) and cell migration and motility (e.g., cell migration, biological adhesion, regulation of cell migration, regulation of cell motility, regulation of locomotion), most of which were also represented 7 days after SE induction in the epileptogenic zone (Figure 3C). These data suggest that early cell signaling processes may be critical for the initiation of pro-epileptogenic remodeling in both the epileptogenic zone and peri-ictal zone. Days 3 and 7 in the peri-ictal zone and days 7 and 14 in the epileptogenic zone shared overlapping enrichment of immune/inflammation-related GO terms (e.g., immune response, response to cytokine, immune effector process, cellular response to cytokine stimulus). Our data therefore suggest that inflammation may also play a role in epileptogenesis, with expression of inflammation-related gene sets occurring in both the epileptogenic zone and peri-ictal zone within the first 14 days after SE induction.

### 2.5. Wnt Pathway Gene Dysregulation during Early Epileptogenesis

Previous work has implicated the Wnt pathway as a factor in neuronal remodeling in the dentate gyrus early after SE induction in this model [30]. Furthermore, Wnt pathway gene changes have also been identified in adult and infantile generalized epilepsy models [22,23,27], suggesting that Wnt pathway gene changes may be relevant in adult focal epilepsy. Due to the representation of cell-signaling-associated GO terms during early epileptogenesis, we specifically examined the transcriptional dysregulation of Wnt pathway genes across the epileptogenic zone and peri-ictal zone. 

**Figure 4:**
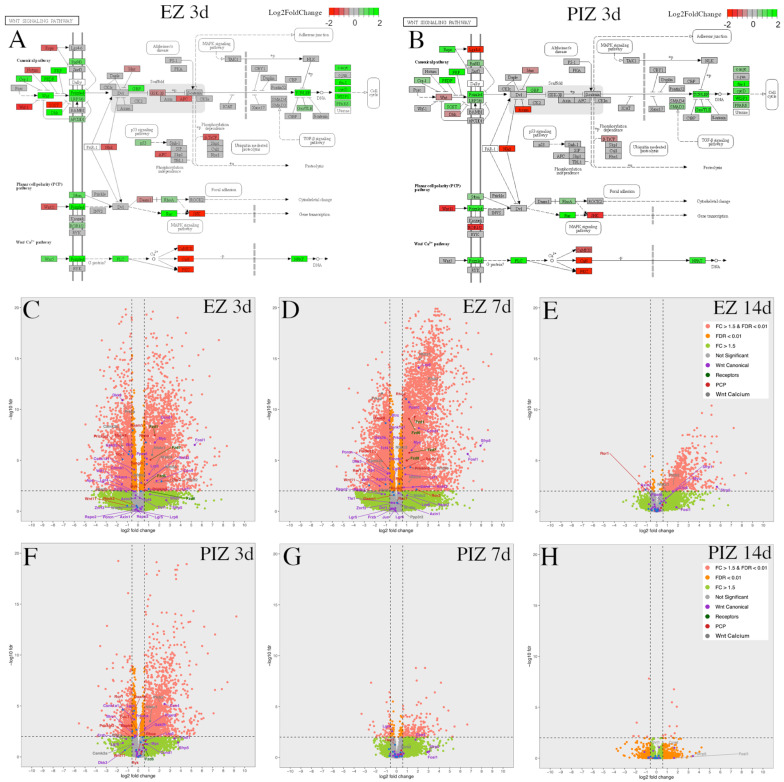
Wnt pathway transcriptional dysregulation in the EZ and PIZ during the first 2 weeks of epileptogenesis. Modified KEGG pathways representing canonical and non-canonical planar cell polarity and calcium pathway Wnt signaling 3 days after SE induction in the (**A**) EZ and (**B**) PIZ. Scale bar represents log2 fold-change in transcription; green color denotes upregulation and red color denotes downregulation. Modified KEGG Wnt signaling pathways for other time-points can be found in Figure S3. Whole transcriptome volcano plots with Wnt signaling mediators individually labeled in the EZ (**C**) 3 days, (**D**) 7 days, and (**E**) 14 days, and in the PIZ (**F**) 3 days, (**G**) 7 days, and (**H**) 14 days after SE induction; dashed lines denote FDR < 0.01 and FC > ±1.5 (equivalent to log2 fold-change > ±0.585). Pink dots represent genes with FDR < 0.01, FC > ±1.5; orange dots represent genes with FDR < 0.01, FC < ±1.5; green dots represent genes with FDR > 0.01, FC > ±1.5; gray dots represent genes with FDR > 0.01, FC < ±1.5. Log2 fold-change values and adjusted *p*-values for these Wnt genes can be found in Table S6.

The modified Kyoto Encyclopedia of Genes and Genomes (KEGG) pathways presented demonstrated Wnt signaling gene expression in the epileptogenic zone (Figure 4A) and peri-ictal zone (Figure 4B) 3 days after SE induction; KEGG pathway representations at other time-points are represented in Figure S3. Upregulation of canonical and non-canonical Wnt signaling mediators and downstream effector genes was observed in both the epileptogenic zone and peri-ictal zone 3 days after SE (Figure 4A,B). In the epileptogenic zone, canonical pathway positive effector genes such as Fzd1 (fold change (FC) 2.99, *p*-adjusted 3.38 × 10^−8^), LRP5 (FC 2.07, *p*-adjusted 2.20 × 10^−4^), Lef1 (FC 1.57, *p*-adjusted 8.35 × 10^−3^), and CTNNB1/beta-catenin (FC 1.23, *p*-adjusted 1.67 × 10^−3^) are noted to be upregulated, and negative regulator genes such as GSK3b (FC − 1.46, *p*-adjusted 4.90 × 10^−6^), Btrc (encoding beta-TrCP [44,45]) (FC 1.42, *p*-adjusted 3.40 × 10^−6^), Wif1 (FC − 2.84, *p*-adjusted 2.13 × 10^−3^), DKK3 (FC − 2.77, *p*-adjusted 1.25 × 10^−11^), and APC (FC − 1.34, *p*-adjusted 1.09 × 10^−2^) were downregulated, suggesting that the canonical Wnt pathway may be activated 3 days after SE in the epileptogenic zone. Non-canonical Wnt pathway genes were also dysregulated, with some genes upregulated, such as Wnt5b (FC 2.41, *p*-adjusted 5.23 × 10^−4^), ROR2 (FC 4.60, *p*-adjusted 1.14 × 10^−3^), RhoA (FC 1.67, *p*-adjusted 1.73 × 10^−7^), and Prickle3 (FC 1.99, *p*-adjusted 6.35 × 10^−3^), and other genes were downregulated, such as Prickle2 (FC − 1.85, *p*-adjusted 2.51 × 10^−6^), JNK (FC − 1.76, *p*-adjusted 1.35 × 10^−6^), PKC (FC − 2.43, *p*-adjusted 4.85 × 10^−11^), and CaMKII (FC − 2.82, *p*-adjusted 3.49 × 10^−7^).

Transcriptional dysregulation of these Wnt pathway genes was followed across the epileptogenic zone (Figure 4C–E) and peri-ictal zone (Figure 4F–H) at each time-point, 3 days, 7 days, and 14 days after SE (Figure 4B–G). Canonical Wnt gene dysregulation was most highly represented in the epileptogenic zone 3 days and 7 days after SE (Figure 4A,C,D). Non-canonical genes were also dysregulated in the epileptogenic zone 3 days and 7 days after SE, with fewer genes significantly altered in expression (fold-change FC > ±1.5, false discovery rate (FDR) < 0.01) than in the canonical Wnt pathway (Figure 4A,C,D). Frizzled receptor genes were upregulated in the epileptogenic zone 3 days and 7 days after SE (Figure 4C,D). In the peri-ictal zone, fewer canonical and non-canonical Wnt genes were dysregulated 3 days after SE than in the epileptogenic zone; none were represented 7 days after SE (Figure 4F,G). Wnt pathway gene differential expression was reduced in both regions 14 days after SE (Figure 4E,H). As Wnt gene dysregulation appeared to reduce progressively over the first 14 days after SE, these data suggest that the first 7 days after SE may represent the therapeutic window for potential Wnt-modulation-based interventions. Differential expression of these genes over time in both the epileptogenic zone and peri-ictal zone is listed with respective log2 fold-change and adjusted *p*-values in Table S6.

### 2.6. Activated-Beta-Catenin Was Upregulated Early after SE Induction in the Epileptogenic and Peri-Ictal Zones

**Figure 5:**
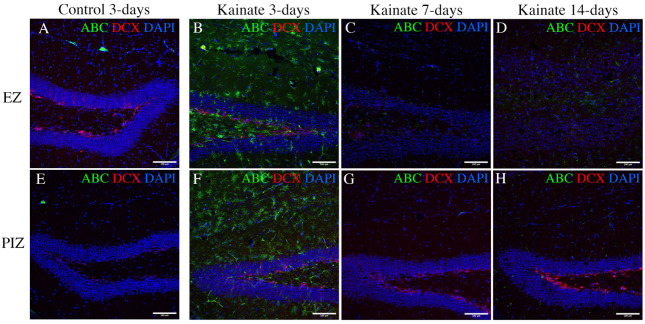
Immunohistochemical evidence for canonical Wnt activation in the hippocampus. Immunofluorescent images demonstrating active beta-catenin (ABC—a marker of canonical Wnt pathway activation) and doublecortin (DCX—a marker of immature dentate granule cells) in (**A**–**D**) the EZ (upper row), and (**E**–**H**) the PIZ (lower row) dentate gyrus. Images demonstrate active beta-catenin expression level (green) (**A**,**E**) in control mice 3 days after saline injection (7 days and 14 days unchanged, data not shown), and in kainate-injected mice (**B**,**F**) 3 days, (**C**,**G**) 7 days, and (**D**,**H**) 14 days after SE. Scale bar 100 µm.

Our transcriptomic analysis suggests that canonical Wnt pathway expression is increased 3 days and 7 days after SE induction in the epileptogenic zone, and in the peri-ictal zone, 3 days after SE. In order to determine whether the transcriptional changes we observed in the Wnt pathway reflect changes in molecular signaling during epileptogenesis, we evaluated the expression of the active form of beta-catenin, the central signaling molecule in the canonical Wnt pathway [33]. We evaluated activated beta-catenin expression through immunohistochemistry in the dentate gyrus 3 days, 7 days, and 14 days after SE induction (Figure 5). As expected, granule cell dispersion was evident in the epileptogenic zone 7 and 14 days after SE, with loss of doublecortin (DCX)-positive dentate granule cells (Figure 5C,D) [11,46,47]. We observed expression of active beta-catenin 3 days after SE induction both in the peri-ictal zone (Figure 5F) and the epileptogenic zone, especially in the subgranular zone and hilus (Figure 5B). Interestingly, active beta-catenin expression diminished at later time-points (Figure 5C,D,G,H), consistent with both our KEGG pathway data (Figure 4A,B) and individual gene expression transcriptional data (Table S6). Quantification of cell-specific expression 3 days after kainate injection demonstrated that 67.4 ± 9.8% of subgranular zone GFAP-positive glial cells and 27.8 ± 7.0% of DCX-positive dentate granule cells co-localized with active beta-catenin in the EZ, and 62.4 ± 11.2% and 27.2 ± 5.1%, respectively, in the PIZ (Figure S4). These results validate our finding of early canonical Wnt activation, as identified in the transcriptomic data.

## 3. Discussion

### 3.1. Transcriptomic Data Implicated a Role for Wnt Pathway Dysregulation in the Seizure Network in Early Epileptogenesis

There is increasing interest in understanding the molecular pathways underpinning pathological remodeling in epileptogenesis. Clinical epilepsy is characterized by the onset of spontaneous recurrent seizures, which often occur in a delayed fashion after exposure to a specific risk factor, such as febrile seizure, infection, tumor, and trauma [6,7,8]. Unfortunately, there are currently no clinical treatments that can be applied to prevent the development of epilepsy after exposure to a known risk factor. Insights into the pathways responsible will be critical for understanding the underlying disease mechanisms and generating novel therapeutic strategies to prevent epileptogenesis.

In the intrahippocampal kainate model of mTLE, the induction of status epilepticus by the injection of intrahippocampal kainate is followed by a latent period prior to the onset of spontaneous recurrent seizures 14 days after injection [14,15,16]. This represents a latent period during which epileptogenesis is believed to occur. Importantly, this latent period is recapitulated in clinical epilepsy. We therefore focused our investigation on this critical period, as the molecular processes that occur may be responsible for epileptogenesis and may represent novel therapeutic targets for seizure prevention. Our choice of time-points has been informed by prior studies. Hansen et al. (2014) performed RNA sequencing of the whole hippocampus using the systemic pilocarpine mouse model of generalized temporal lobe epilepsy [25]. Time-points were selected across various periods of epilepsy, with 12 h representing acute SE, 10 days considered the latent period, and 6 weeks representing chronic epilepsy. As might be expected, the investigators found that the three time-points differed in transcriptomic profile as they represent different phases of pre-clinical model epilepsy development. Interestingly, they found increased similarity between the 12 h acute SE and 6-week chronic phase, which the authors conclude may have been due to ictal activation-related changes, with the 10-day time-point representing a unique phase specific to epileptogenesis. These insights corroborate our choice of time-points within the latent epileptogenic period [16,25,48].

We focused our study on the hippocampal dentate gyrus as it undergoes extensive remodeling in epileptogenesis and is believed to be responsible for the development of spontaneous recurrent seizures in mTLE [10,11,13,43,49]. The dentate gyrus has been the focus of other studies, however, using systemic chemoconvulsant/generalized epilepsy model systems [22,23,26]. Uniquely, our use of a focal pre-clinical model of mTLE and transcriptomic examination of the bilateral dentate gyri allows for investigation of different regions of the seizure network, including the epileptogenic zone and peri-ictal zone, as it has been shown previously that both the ipsilateral and contralateral hippocampi undergo pathological remodeling events [30] and generate independent seizures [41]. Our study therefore uniquely provides insights into their relative contributions to epileptogenesis. This may enhance clinical translation, as there is increasing awareness of the role of the seizure network in human clinical epilepsy, with network nodes being targeted therapeutically and being believed to be responsible for seizure recurrence after epilepsy surgery [19,20,21,50].

Our data interestingly demonstrated shared patterns of transcriptomic dysregulation in the epileptogenic zone and the peri-ictal zone during the first week of epileptogenesis. These included enrichment of cell signaling, motility, and proliferation functional categories, as might be expected given the pathological remodeling of dentate granule cell neurons observed in the dentate gyrus during epileptogenesis. Interestingly these processes declined within 14 days of SE induction in both the epileptogenic zone (EZ) and peri-ictal zone (PIZ). Since extensive remodeling is observed in the epileptogenic zone and peri-ictal zone 2 weeks after seizure induction [30], it is possible that these early transcriptional changes may contribute to epileptogenic remodeling. Our finding that both the ipsilateral EZ and contralateral PIZ demonstrate shared transcriptional events corroborates with the description of the contralateral hippocampus as part of the seizure network as the contralateral hippocampus undergoes pathological remodeling and generates seizures after ipsilateral kainate injection [28,30,41]. These data, in conjunction with previously published findings, also have implications for the use of the contralateral hippocampus for baseline control measurements.

Our investigation of serial time-points highlights the dynamic nature of transcriptional dysregulation events after seizure induction. Our data demonstrate that cell-signaling events and Wnt signaling were increased 3 days after seizure induction and returned to baseline within 7 days. Moreover, by contrast, inflammation in the EZ was most apparent 7–14 days after seizure induction. If these processes are to be targeted for epilepsy treatment, accurate timing may be critical. Furthermore, sampling outside of this period may not capture these early gene changes. Indeed, in our data, immune processes followed a different time-course in the epileptogenic zone and peri-ictal zone. In the epileptogenic zone, immune representation was delayed, beginning 7 days after SE, and was sustained to 14 days after SE. Conversely, in the peri-ictal zone, immune processes were represented 3 days and 7 days after SE and were no longer represented 14 days after SE. These findings also suggest that inflammation may play a role in altering the neuronal phenotype and warrant further investigation in future studies.

In our previous work, we investigated the role of the Wnt pathway in epileptogenesis utilizing this model, finding that specific Wnt pathway ligands were differentially expressed in the epileptogenic zone and peri-ictal zone at a single time-point 3 days after SE induction [30]. Data from our current study corroborate our previous findings, with upregulation of Wnt5b in the epileptogenic zone, Wnt7b upregulation across both the epileptogenic zone and peri-ictal zone, and Wnt9a downregulation across both the epileptogenic zone and peri-ictal zone, providing evidence for the robustness of the model. The whole transcriptome data in the current study builds on our previous work and suggests that canonical Wnt signaling mediators are upregulated in the epileptogenic zone during epileptogenesis, either in a causative capacity or reactive to SE. We also observed expression of active beta-catenin protein by immunofluorescence in the epileptogenic zone and peri-ictal zone 3 days after SE induction; while this correlated with our gene expression data for beta-catenin in the epileptogenic zone, it did not in the peri-ictal zone. However, beta-catenin is regulated both by expression and also by phosphorylation status and degradation by GSK3b [33]; therefore, transcription level of beta-catenin alone may not fully reflect signaling activity and activity of downstream genes. Direct assessment for active beta-catenin can provide a more accurate picture, as demonstrated in our immunohistochemistry and KEGG pathway data.

Differential gene expression in the Wnt pathway has been described in other animal pre-clinical epilepsy models, corroborating our findings. Canto et al. (2021) investigated transcriptomic and proteomic changes 15 days after SE, during the epileptogenic period, using the rat systemic pilocarpine bilateral temporal lobe epilepsy model; tissues were separated into dorsal and ventral dentate gyrus and CA3 sub-regions. The investigators observed a decrease in neuronal markers and increased microglial markers in the DG and CA3 sub-regions, and specifically implicated NMDA excitotoxicity, calcium/calmodulin-dependent protein kinase (CaMK), and LRRK2/Wnt signaling [22]. Interestingly, this study reported increased expression of CaMKs and LRRK2, which we observed in our data, as well as specific enrichment of the pathway “adult neurogenesis in the subventricular zone” in the dentate gyrus, which they concluded to be mediated by LRRK2, Wnt, and CDK5. LRRK2 is a Wnt signaling scaffold protein that has been implicated in the pathogenesis of Parkinson’s disease [51] and mediates canonical Wnt signaling [52].

Other Wnt-related processes were also upregulated in the DG, including nuclear factor kappa b (NFKB) and transforming growth factor-beta (TGF-beta) signaling. TGF-beta signaling has been observed after neurological injury and has been shown to modulate the extracellular matrix, specifically affecting peri-neuronal nets surrounding inhibitory neurons; TGF-beta has therefore been mechanistically linked to post-traumatic epilepsy [53]. Supporting this hypothesis, blockade of TGF-beta signaling by losartan prevented seizures in an albumin-induced epilepsy model [54]. Serum albumin release into the brain after blood–brain barrier disruption has been specifically implicated in upregulating TGF-beta signaling and is associated with astrocyte and microglial activation as well as a pro-inflammatory response including NFKB activation [55]. Indeed, given the relationship between inflammation and NFKB signaling [56], NFKB has also been implicated in regulating seizure threshold [57] and may potentiate neuronal death in temporal lobe epilepsy [58,59]. Additional pathways may also, therefore, contribute to the development of epilepsy.

Given the variability in pre-clinical epilepsy models, Dingledine et al. (2017) utilized a number of rat pre-clinical generalized epilepsy models, including systemic pilocarpine, systemic kainite, and electrical kindling models, and extracted the mid-layer of the right dentate gyrus for RNA sequencing. Of the 72 dysregulated genes common to all models, 19 genes were related to Wnt signaling. These genes were ABCD2 [60], C1s [61], CBFB [62], CRIM1 [63], Ext1 [64], FAM129B [65], FAT1 [66], GDF10 [67], GPC3 [68], KHDRBS3 [69], KLF15 [70], Lox [71], MMP9 [72], NTF3 [73], SS18 [74], SSBP3 [75], TRH [76], Wls [77], and ZMIZ1 [78]. Of these genes, 17 are represented in our transcriptomic data, highlighting the overlap in molecular signaling identified in their study and ours. The authors did not, however, specifically implicate the Wnt pathway. Their methods investigated the mid-layer of the dentate granule cell layer by laser microdissection. While specific for the dentate, this method excluded the region adjacent to the hilus containing immature dentate granule cells, which are known to undergo remodeling during epileptogenesis [12,30,79]. Our histological examination demonstrated that, within the dentate gyrus, active beta-catenin (a marker of canonical Wnt signaling activation) was predominantly expressed in this region adjacent to the subgranular zone and less so in the mid-dentate gyrus. This provides some suggestion as to why Wnt signaling may have been less represented in their study overall. Interestingly, we observed active beta-catenin staining in subgranular zone glial cells and immature dentate granule cells, suggesting that in early epileptogenesis, canonical Wnt signaling is increased over baseline levels [37,38], an observation also made in other studies [27,29,80]. This suggests that canonical Wnt signaling may contribute to aberrant development of dentate granule cells during epileptogenesis.

Work by Theilhaber et al. (2013) also implicated Wnt signaling in pre-clinical epileptogenesis. Using a neonatal rat pup hypoxia model of generalized epilepsy, the whole hippocampus and cortex were analyzed by RNA sequencing; the investigators observed increased expression of canonical Wnt signaling molecule genes such as beta-catenin, LRP6, and Dvl3, as well as inhibitors such as GSK3b, sFRP2, and Wif1. Notably, in this model, hippocampal cell death is not observed after hypoxic seizure, which differs from kainate-based models [81,82]. Nonetheless, the findings of Wnt dysregulation across numerous different pre-clinical epilepsy models potentially suggest that the Wnt pathway may be relevant in the pathogenesis of epilepsy. Indeed, the Wnt pathway has been implicated experimentally in several rat epilepsy models. Using a systemic pilocarpine model, mossy fiber sprouting into the CA3 region of the hippocampus correlated with N-cadherin and beta-catenin expression, suggesting that the canonical Wnt signaling plays a role in pathological axonal sprouting and path-finding in epilepsy [39]. Similarly, using an electroconvulsive epilepsy model, increased expression of Wnt2 and beta-catenin was observed in the hippocampal subgranular zone during the first 48 h after seizure induction, supporting our observation that canonical Wnt signaling is upregulated in early epileptogenesis [80]. In a systemic kainate rat epilepsy model, seizure induction was associated with a reduction in canonical Wnt signaling mediators Wnt1, beta-catenin, Dvl1, and phospho-GSK3b (inactive Wnt inhibitor) 72 h after injection; anti-epileptic drug treatment (carbamazepine) increased expression of these canonical Wnt signaling mediators and was associated with reduced neuronal death, as well as reduced glutamate excitotoxicity, implicating canonical Wnt signaling as a potential neuroprotective signaling pathway [31]. Supporting a neuroprotective role for canonical Wnt signaling, in a chronic systemic pilocarpine rat epilepsy model, epileptic animals were found to have reduced synaptic spine density in the dentate gyrus and CA1 regions of the hippocampus, as well as long-term potentiation at CA1 Schaffer collaterals, which was associated with reductions in canonical Wnt signaling; intermittent hypobaric hypoxia rescued these deficits in a canonical-Wnt-signaling-dependent manner as DKK1 infusion (canonical Wnt inhibitor) blocked its therapeutic effect [32]. Competing with these observations, however, is the finding that HBP1 knockout mice (a known repressor of Wnt signaling [83]) developed spontaneous seizures, which the investigators attributed to increased Wnt and mTor signaling within the first 5 days after systemic chemoconvulsant injection [29].

As a result, the specific role of Wnt pathway dysregulation in epileptogenesis remains to be fully elucidated. Given the complexity of the signaling pathways, including mixed changes in expression of the non-canonical pathways and inhibitor molecules evidenced in our data, it is possible that the role of Wnt pathway dysregulation in epileptogenesis may be temporally defined, as it is during hippocampal neurogenesis [84]. Furthermore, different Wnt pathways and signaling mediators may play different roles in the formation of the epileptogenic zone and peri-ictal zone. Indeed, Wnt signaling has been reported as both protective and deleterious in rat generalized epilepsy models; the effect of network/region-specific changes remain to be fully elucidated [29,31,32,39,80].

### 3.2. Study Limitations

There are limitations of our study that ought to be considered in interpretation of the data. The pre-clinical focus uniquely allows access to mammalian hippocampal tissues at early timepoints during epileptogenesis, which cannot be routinely accessed in human epilepsy patients as time to presentation for epilepsy surgery can be many years from first presentation [85]. Variation in pre-clinical models and their concordance with clinical epilepsy must therefore be considered; the presence of pathological changes in the dentate gyrus of epileptic mice and humans is now well documented, with shared histological features such as granule cell dispersion, hippocampal sclerosis, aberrant migration, and dendrite formation [10,12,13,86,87,88,89,90]. These lend biological credibility to pre-clinical animal models; however, human studies are still needed to validate pre-clinical findings and generate hypotheses for development of novel therapeutics targeting epileptogenesis. Human tissue studies have been reported, and though interpretation of findings is limited by the use of cadaveric and allogenous tumor-associated tissues as controls, Wnt pathway genes such as casein kinase have been observed in human mTLE [24,91]. Attempting to increase the fidelity of pre-clinical models to human clinical epilepsy is therefore of value to improve translation. Our utilization of a focal model of mesial temporal lobe epilepsy as a model for human mTLE aims to address this; however, the use of kainate injection may not accurately recapitulate the typical etiology in humans. Nonetheless, insights can be made into the epileptogenic process.

Given the role of the dentate gyrus in epileptogenesis, our study focused on micro-dissected dentate gyrus tissue, as described previously [30], as opposed to laser microdissection studies that focus on specific sub-regions of the dentate gyrus [23], and largely excluded immature dentate granule cells, the principal cell type aberrant in pre-clinical TLE [12,79]. While the dissected tissue does include the hilus region, the whole dentate is sampled and thereby includes immature and mature neurons as well as associated non-neuronal cell types, thereby reflecting the overall signaling environment in the dentate gyrus. Another limitation of our study, therefore, is that in using bulk RNA-seq for transcriptomic analysis, we cannot uncover cell-type-specific contributions. The specific contributions of individual cell types to the signaling environment will require further cell-specific studies.

## 4. Materials and Methods

### 4.1. Animal Husbandry and Experimental Design

Experiments were performed using adult wild-type C57Bl6/J mice (Jax #000664) of 8–10 weeks of age. Mice were housed in group cages with a maximum of 5 animals per cage, with free access to food and water ad libitum, according to local IACUC guidelines. All animal procedures were performed in accordance with the Indiana University animal care committee’s regulations. For these experiments, mice received either unilateral intrahippocampal saline (control) or unilateral intrahippocampal kainate by stereotactic injection. Brains were extracted 3 days, 7 days, and 14 days after intrahippocampal injection, and dentate gyri were anatomically dissected from both hemispheres for RNA sequencing. Each of the 6 groups included 4 animals, 2 male and 2 female. Samples were analyzed separately and not pooled; our use of 4 independent animals for each group meets or exceeds sample numbers described in other studies [25,27,92].

### 4.2. Intrahippocampal Kainate-Induced Status Epilepticus

Mice were induced and maintained under anesthesia with isoflurane by spontaneous respiration. The head was secured with ear bars in a stereotactic apparatus (Stoelting, Wood Dale, IL, USA), the head was shaved, and the scalp was sterilized with alternating ethanol and betadine. A single midline incision was made, and stereotactic coordinates were obtained relative to bregma. A single burr hole was placed at ML + 1.8, AP − 2.1, and the debris was cleared with sterile saline injection. The injection needle (Hamilton, Reno, NV, USA) was slowly advanced to DV -1.9 from the skull. A total of 85 nl of sterile normal saline or kainate (18.7 nM in normal saline, MilliporeSigma, Burlington, MA, USA) was delivered over 1 min using the Quintessential Stereotactic Injector (Stoelting, Wood Dale, IL, USA); the needle was retained in position for 2 min to prevent reflux prior to slow removal. The skin was closed using VetBond dermal glue (3M, Saint Paul, MN, USA), and the animal was recovered in a warmed chamber. Seizures were scored for 2 h after injection by modified Racine scale [93]; stages 1 and 2—freezing, head nodding, mastication; stage 3—forelimb clonus; stage 4—rearing; stage 5—rearing and falling; stage 6—“popcorn”-type seizure. Induction of status epilepticus (SE) is well described after intrahippocampal kainate injection; therefore, seizure induction was confirmed behaviorally without the use of EEG, as described previously [11,40,79,94,95,96]. Only mice that underwent at least one observed Racine 3–6 seizure were included in the epilepsy group; all animals that received kainate incurred a Racine 3–6 seizure within the 2 h observation period, and no saline-injected mice demonstrated behavioral seizure activity. Mice received food saturated with pediatric Tylenol (3.2 mg/mL) for 24 h after surgery for analgesia, as required by local IACUC guidelines, and soft food daily until tissue extraction. Tylenol is not an anti-inflammatory drug [97,98], and non-steroidal anti-inflammatory drugs (NSAIDs) were not administered.

### 4.3. RNA Extraction and Library Preparation

Brains were extracted 3 days, 7 days, and 14 days after intrahippocampal injection. The dentate gyri were anatomically microdissected in an RNAse-free environment. The ipsilateral and contralateral dentate gyri were isolated separately and hemisected into dorsal and ventral parts as described previously [30]; dorsal dentate gyri were used for transcriptomic analysis. The ipsilateral dentate gyrus has been shown to undergo extensive neuronal loss, gliosis, and remodeling of immature dentate granule cells [43,47,99]; the contralateral dentate gyrus also undergoes remodeling and generates independent seizures [30,41]. Therefore, as published previously [30], the dorsal ipsilateral dentate gyrus tissue is denoted to represent the epileptogenic zone (EZ), synonymous with the ictal zone, and the dorsal contralateral dentate gyrus tissue is denoted to represent the peri-ictal zone (PIZ). Tissues were preserved in RNALater (AM7020, Invitrogen, Waltham, MA, USA) for 24 h at 4 °C, mechanically macerated (Kimble-Chase, Vineland, NJ, USA), and stored at −80 °C until all tissues were collected. RNA extraction was performed for all samples simultaneously, utilizing high-throughput automation. Samples were prepared for extraction using the standard protocol for lipid-rich samples described in the QIAsymphony RNA Handbook (November 2020 edition). Tissue disruption and homogenization was carried out using a Qiagen Tissuelyser II. RNA extraction was performed using a QIAsymphony SP (protocol: RNA_CT_800_V7, kit: QIAsymphony RNA Kit (192), catalog no. 931636). In brief, RNA was separated from lysates through binding to magnetic particles, DNA was removed by RNase-free Dnase treatment, and RNA was eluted into 100 µL of RNase-free water and analyzed using an Agilent 4200 TapeStation. All samples received RNA integrity scores over 7.5. Poly-A selected libraries were prepared with the Illumina TruSeq Stranded mRNA Library Preparation Kit protocol (catalog no. 20020595). The libraries were analyzed by Agilent 4200 TapeStation, and pooled. The pooled libraries were loaded on a NextSeq 500 High Output v2.5 run with a 75-cycle sequencing module (catalog no. 20024906) to generate paired-end reads. The demultiplexing of the reads was performed with bcl2fastq version 2.20.0 (Illumina, San Diego, CA, USA).

### 4.4. RNA Sequencing and Bioinformatic Analysis

A total of 48 poly-A selected RNA-seq libraries were sequenced to an average depth of 27.8 million read pairs. On average, 24.5 million read pairs were mapped as properly paired reads (88%) per sample. Of these 24.5 million read pairs, 21.2 million could be assigned to known features (87%). NextSeq reads were trimmed using fastp (version 0.20.1) with parameters “-l 17 --detect_adapter_for_pe -g -p” [100]. The resulting reads were mapped against GRCm39 using HISAT2 (version 2.2.1) with parameters “--rna-strandness F” [101]. HISAT uses Bowtie2, which is based on the Burrows–Wheeler transform algorithm for sequence alignment and allows for mapping across exon junctions [102]. Read counts for each gene were created using featureCounts from the Subread package (version 1.6.4, Bioconductor, Boston, MA, USA) with the parameters “-O -M --primary -p --largestOverlap -s 2 -B” and Gencode M28 as the annotation [103,104]. Differential expression analysis was performed using the DESeq2 package (version 1.32.0) in R/Bioconductor (R version 4.1.1, Bioconductor, Boston, MA, USA) [105]. Functional analysis was performed using The Database for Annotation, Visualization and Integrated Discovery (DAVID) version2022q1 (FIS, Jacksonville, FL, USA) [106,107]. DAVID is an online functional annotation tool used for gene enrichment analysis. Gene lists for each timepoint were selected using fold-change (FC) > 1.5 and false discovery rate (FDR) < 0.01, then analyzed through DAVID web services using custom perl scripts. Gene ontology (GO) terms (GOTERM_XX_FAT), Kyoto Encyclopedia of Genes and Genomes (KEGG) pathways, and BioCarta Pathways were used for the functional enrichment analysis [108,109,110,111,112,113]. KEGG and BioCarta are online databases of chemical and biological networks used to ascribe pathway enrichment in large datasets. GO terms are formal representations of biological domains and functions. Genes with FDR < 0.01 and FC < ±1.5 were also considered in data analysis, specifically when considering overlapping gene expression groups in Figure 2 (labeled as Sig < 1.5 FC), in order to include significant genes with less than ± 50% alteration in expression level that may nonetheless be biologically significant.

### 4.5. Immuno-Histochemistry

Animals were transcardially perfused with saline and 4% paraformaldehyde (Sigma-Aldrich, St. Louis, MI, USA) in 0.1 M PBS. Brains were extracted and placed in 4% PFA for 24 h, then dehydrated in 30% sucrose prior to sectioning. Coronal sections of the brain were obtained using a cryostat (Leica Microsystems, Wetzlar, Germany) at 40 µm slice thickness. Slices were blocked and permeabilized in PBS containing 5% normal goat serum (Cell Signaling, Danvers, MA, USA) and 0.4% Triton-R X-100 (Alfa Aesar, Haverhill, MA, USA) for 1 h. Primary antibodies were applied at 1:500 overnight at 4 °C, and Alexa-Fluor-conjugated secondary antibodies (Life Technologies, Carlsbad, CA, USA) were applied at 1:1000 overnight at 4 °C. DAPI was applied at 1:10,000 dilution for 45 min, and sections were mounted with VectaShield HardSet (H-1400, Vector Laboratories, Newark, CA, USA). Antibodies included activated beta-catenin (05-665, MilliporeSigma, Burlington, MA, USA) and doublecortin (AB2253, MilliporeSigma). Images were obtained by confocal microscopy (Olympus FV1000, 20×, 405 nm 5%, 473 nm 3%, 559 8%), and all images were acquired at identical settings.

## 5. Conclusions

In conclusion, our study investigated transcriptomic changes across a focused period during epileptogenesis, the latent period between status epilepticus and the development of spontaneous recurrent seizures. We differentiated between different regions within the seizure network, utilizing the intrahippocampal kainate model of focal mesial temporal lobe epilepsy to examine the epileptogenic zone and the peri-ictal zone. We specifically examined Wnt pathway dysregulation and found canonical Wnt pathway activation in the epileptogenic zone and peri-ictal zone early in the latent period. Whether the canonical Wnt pathway is acting in a causative or reactive manner remains to be determined in future studies. Many questions remain: Can these pathways be modulated to alter the course of epileptogenesis? Do various clinical presentations and pre-clinical models of epilepsy share common signaling pathways? Is there re-emergence of various signaling pathways during breakthrough clinical seizures or during recurrence after epilepsy surgery? Our database can be interrogated to determine the involvement of other molecular pathways in the formation of the seizure network. There remains a need for improved graphic representation, data integration amongst studies, and defined epilepsy-specific gene-sets for data analysis interpretation. With these, a more integrated view of epileptogenesis may be achieved.

## Data Availability

Associated materials and protocols are available upon request. The RNA-seq datasets are accessible through NCBI Gene Expression Omnibus at GEO series accession number GSE213393.

## Supplementary Materials

The following supporting information can be downloaded at https://www.mdpi.com/article/10.3390/ijms231912030/s1.
