# Supplementary material for: Transcriptome Profiling of the Hippocampal Seizure Network Implicates a Role for Wnt Signaling during Epileptogenesis in a Mouse Model of Temporal Lobe Epilepsy
Source: Int J Mol Sci. 2022 Oct 10;23(19):12030. doi: 10.3390/ijms231912030 (PMC9569502; doi:10.3390/ijms231912030)
Supplement: Supplementary file 1 [file ijms-23-12030-s001.zip › Supplementary Figures.pdf]

Supplementary Figures:

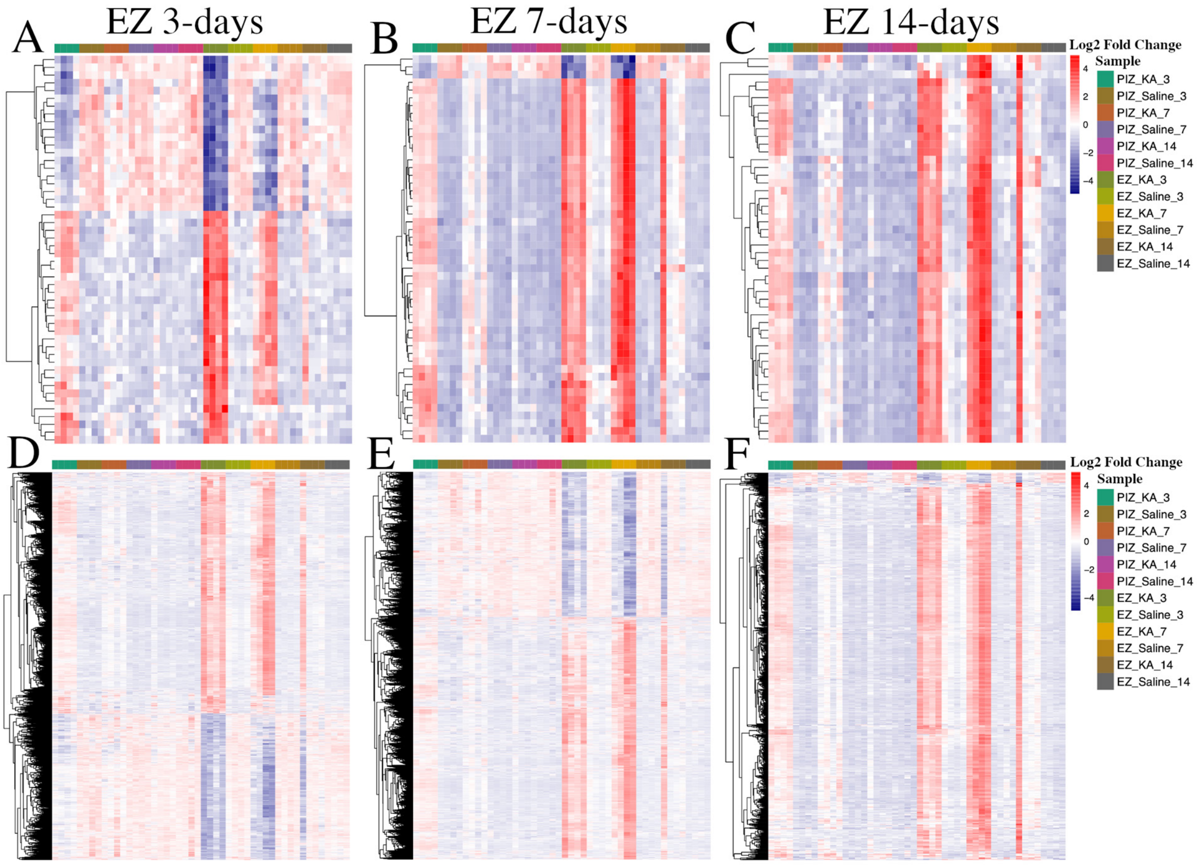

Figure S1. Hierarchical cluster analysis of transcriptional dysregulation in the EZ and PIZ during the first 2 weeks of epileptogenesis. Heatmaps of (A–C) the top 50 dysregulated genes and (D–F) whole transcriptome, FDR < 0.01 FC > ± 1.5, in the epileptogenic zone and peri-ictal zone (A,D) 3 days, (B,E) 7 days, and (C,F) 14 days after SE induction.

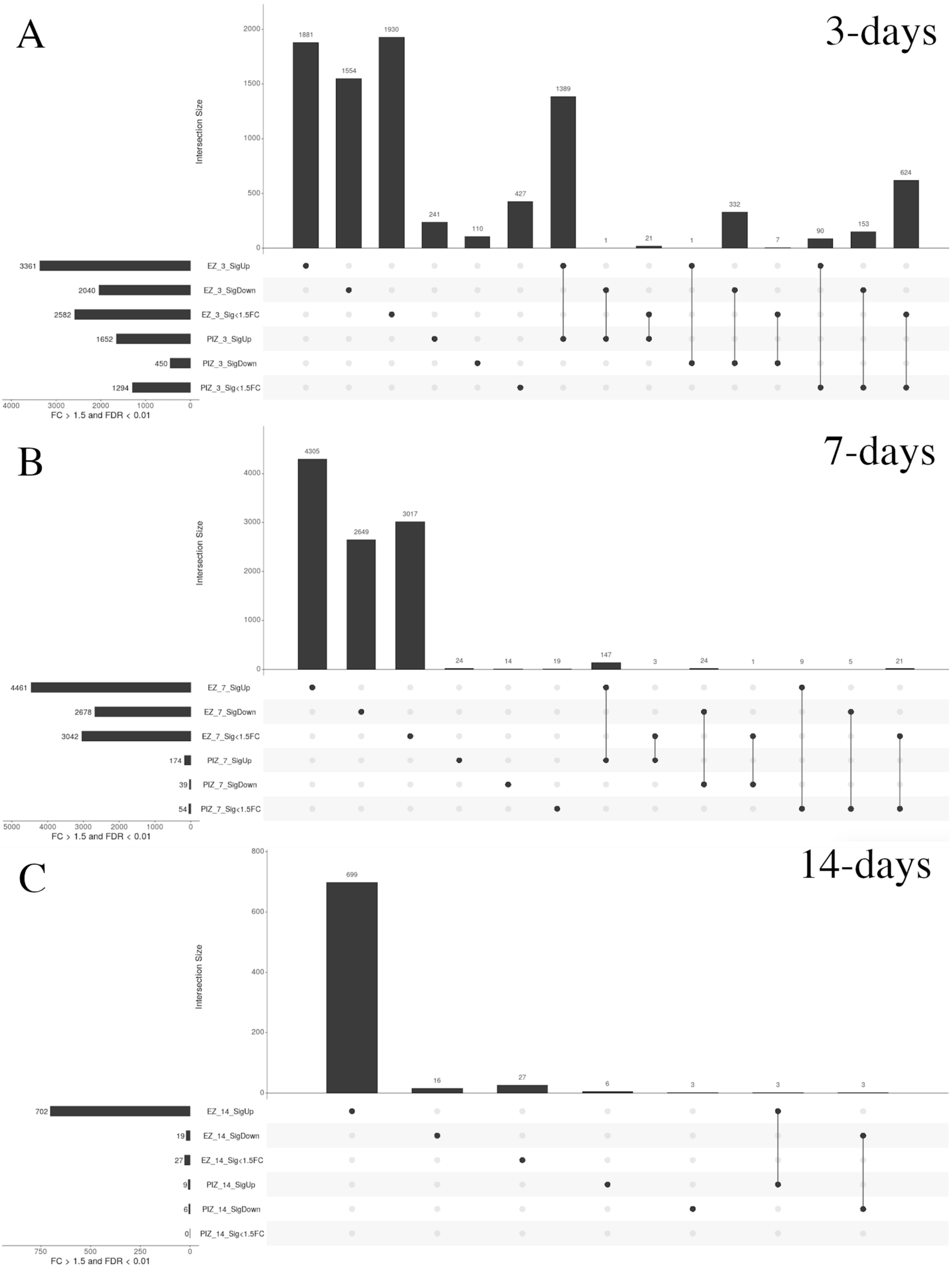

A
3-days
Intersection Size
1881
1554
1930
241
110
427
1389
1
21
1
332
7
90
153
624
3361 EZ_3_SigUp
2040 EZ_3_SigDown
2582 EZ_3_Sig<1.5FC
1652 PIZ_3_SigUp
450 PIZ_3_SigDown
1294 PIZ_3_Sig<1.5FC
FC > 1.5 and FDR < 0.01
B
7-days
Intersection Size
4305
2649
3017
24
14
19
147
3
24
1
9
5
21
4461 EZ_7_SigUp
2678 EZ_7_SigDown
3042 EZ_7_Sig<1.5FC
174 PIZ_7_SigUp
39 PIZ_7_SigDown
54 PIZ_7_Sig<1.5FC
FC > 1.5 and FDR < 0.01
C
14-days
Intersection Size
699
16
27
6
3
3
3
702 EZ_14_SigUp
19 EZ_14_SigDown
27 EZ_14_Sig<1.5FC
9 PIZ_14_SigUp
6 PIZ_14_SigDown
0 PIZ_14_Sig<1.5FC
FC > 1.5 and FDR < 0.01

Figure S2. UpSet plots depicting overlap in differentially expressed genes across the EZ and PIZ, (A) 3 days, (B) 7 days, and (C) 14 days after SE induction.

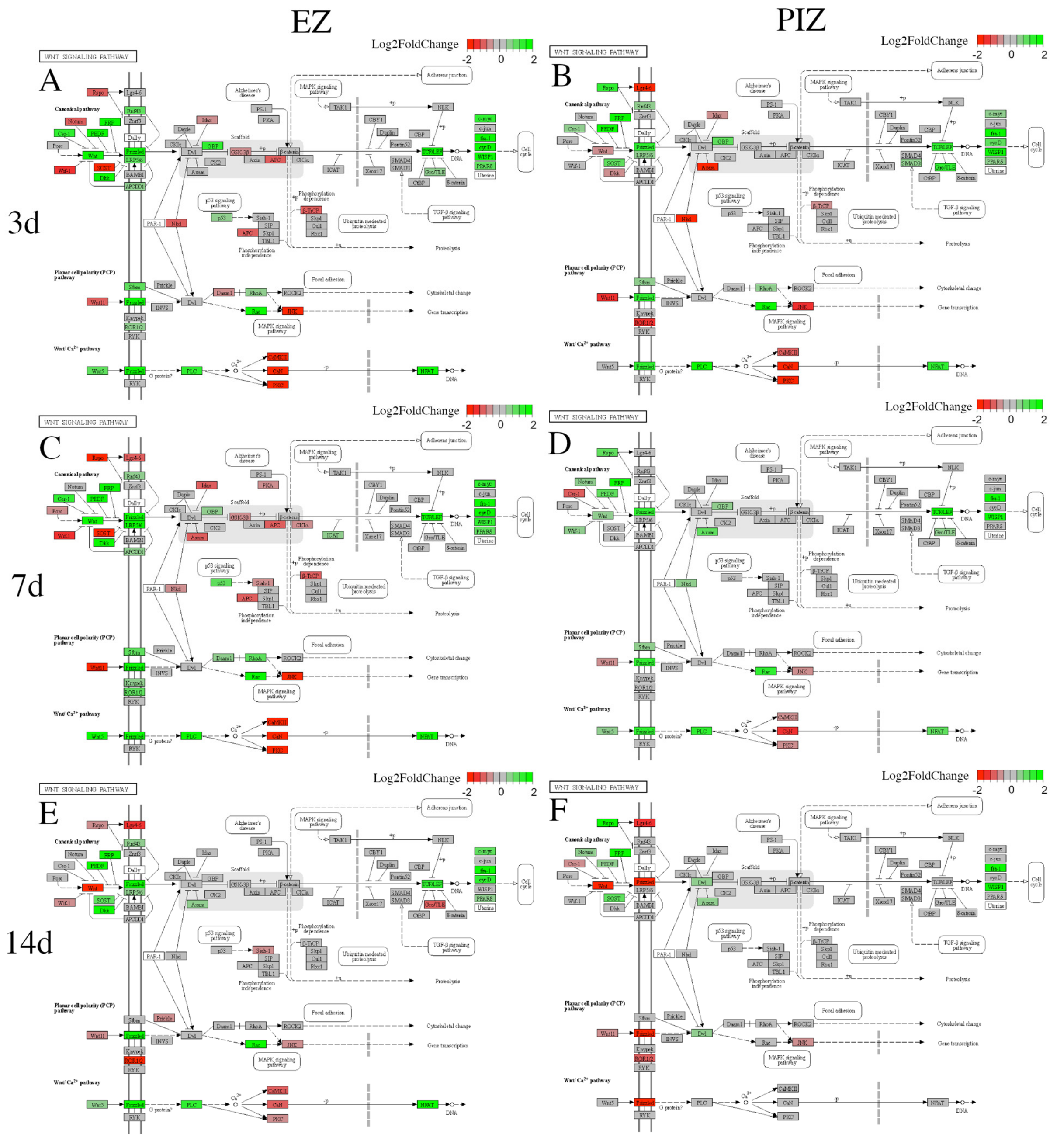

Figure S3. Modified KEGG pathway diagrams depicting canonical and non-canonical PCP and calcium Wnt signaling pathways in the EZ and PIZ after SE induction. Wnt signaling at 3 days depicted in (A) EZ and (B) PIZ, at 7 days in (C) EZ and (D) PIZ, and at 14 days in (E) EZ and (F) PIZ.

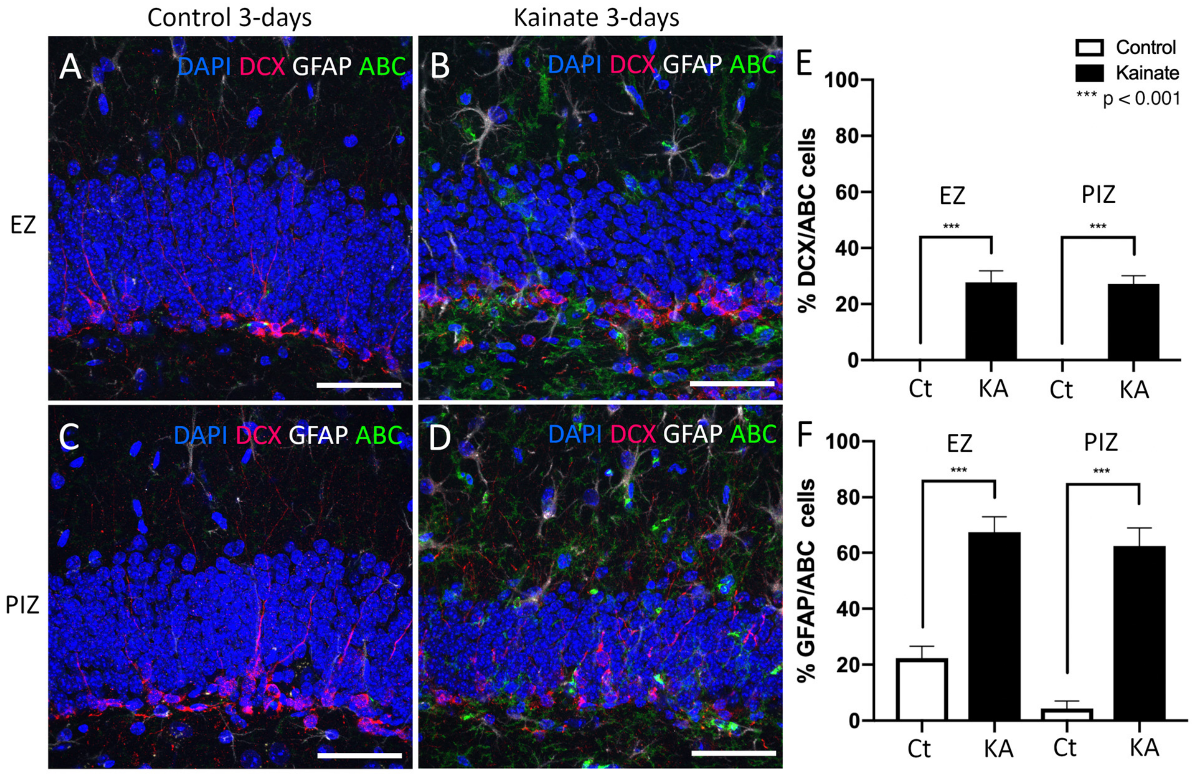

Figure S4. Cellular localization of active beta-catenin 3 days after kainate. Immunofluorescent images demonstrate active beta-catenin (ABC), doublecortin (DCX), and glial fibrillary acid (GFAP) expression in the dentate gyrus at 3 days in (A) control EZ, (B) kainate EZ, (C) control PIZ, and (D) kainate PIZ. Histograms demonstrating quantification of co-localization for (E) DCX and ABC, and (F) subgranular zone GFAP and ABC in the EZ and PIZ. Scale bar 50 µm, *** $p < 0.001$ (Student's *t*-test).

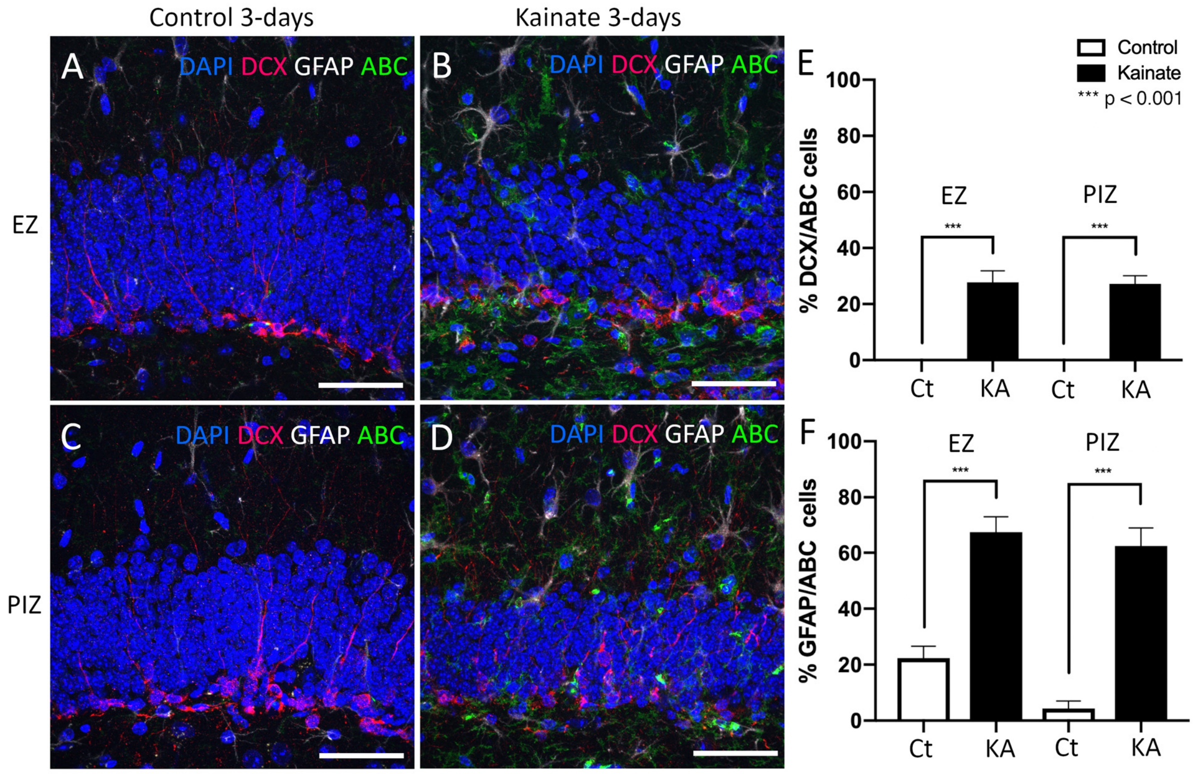

Figure S5. Cellular localization of active beta-catenin 3-days after kainate. Immunofluorescent images demonstrate active-beta-catenin (ABC), doublecortin (DCX) and glial fibrillary acid (GFAP) expression in the dentate gyrus 3-days in A) Control EZ, B) Kainate EZ, C) Control PIZ, D) Kainate PIZ. Histograms demonstrating quantification of co-localization for E) DCX & ABC and F) subgranular zone GFAP & ABC in the EZ and PIZ. Scale bar 50µm, *** $p<0.001$ (Student's t-test).
